# Supplementary material for: Dynamic ParB–DNA interactions initiate and maintain a partition condensate for bacterial chromosome segregation
Source: Nucleic Acids Res. 2023 Oct 18;51(21):11856–75. doi: 10.1093/nar/gkad868 (PMC10681803; doi:10.1093/nar/gkad868)
Supplement: gkad868_Supplemental_Files [file gkad868_supplemental_files.zip › Tisma et al_Supplementary file.pdf]

Supplementary Figures for

Dynamic ParB-DNA interactions initiate and maintain a partition condensate for bacterial chromosome segregation

Miloš Tišma[1], Richard Janissen[1], Hammam Antar[2], Alejandro Martin Gonzalez[1], Roman Barth[1], Twan Beekman[1], Jaco van der Torre[1], Davide Michieletto[3,4], Stephan Gruber[2], Cees Dekker[1†]

[†] Corresponding author: C.Dekker@tudelft.nl

[1] Department of Bionanoscience, Kavli Institute of Nanoscience Delft, Delft University of Technology; Delft, Netherlands.

[2] Department of Fundamental Microbiology (DMF), Faculty of Biology and Medicine (FBM), University of Lausanne (UNIL); Lausanne, Switzerland.

[3] School of Physics and Astronomy, University of Edinburgh, Edinburgh, UK.

[4] MRC Human Genetics Unit, Institute of Genetics and Cancer, University of Edinburgh, Edinburgh, UK.

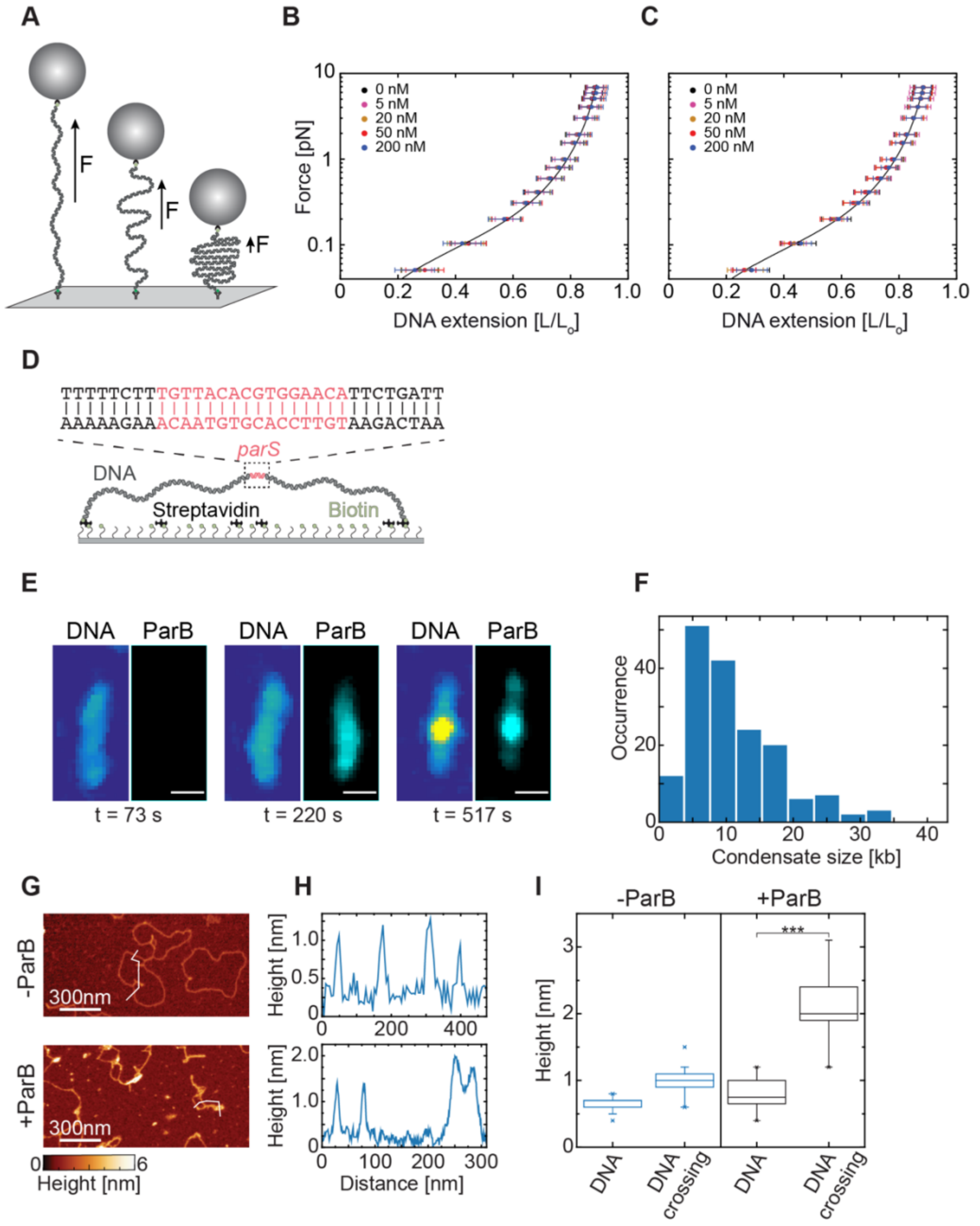

Figure S1. ParB proteins do not condense the DNA in the absence of CTP or without a *parS* site.

(A) Representation of experimental MT assay where the force is gradually lowered and the bead tethered to 12 kbp linear DNA moves towards the surface in presence of different ParB concentrations. **(B)** Average DNA extension (mean±SD) at lowering forces (N; 0 nM: 61, 5 nM: 61, 20 nM: 60, 50 nM: 60, 200 nM: 57) in the absence of a *parS* site on the DNA. (**C**) Average DNA extension (mean±SD) at lowering forces (N; 0 nM: 16, 5 nM: 15, 20 nM: 15, 50 nM: 17, 200 nM: 11) in the absence of CTP nucleotide. Black lines in (B) and (C) represent a WLC fit to the 0 nM ParB data. **(D)** Schematic representation of DNA_{parS} tethered with both ends to a glass surface via streptavidin-biotin interactions (see Methods). (**E**) Representative fluorescence images of DNA_{parS} and $ParB^{TMR}$ signals at three time points during the experiment: (left) before ParB is present, (middle) at the moment that ParB covers the DNA_{parS}, and (right) after the DNA_{parS}

is visibly condensed by ParB molecules. t = 0 s represents the imaging start time after the buffer exchange to the imaging buffer containing ParB protein (**F)** The size of ParB-induced DNA condensates on the 42 kbp DNA_{parS} molecule (N = 161) **(G)** Zoomed AFM image of DNA_{parS} molecules in the presence and absence of ParB (7 nM) from Fig. 1G, I. White line represents the path over which height profile was analyzed. **(H)** Height profiles over the lines presented in (G). **(I)** Height of the DNA_{parS} structures in the absence of ParB molecules (7 nM), both at single strands and at the section where two DNA strands cross each other (p: *** <0.001)

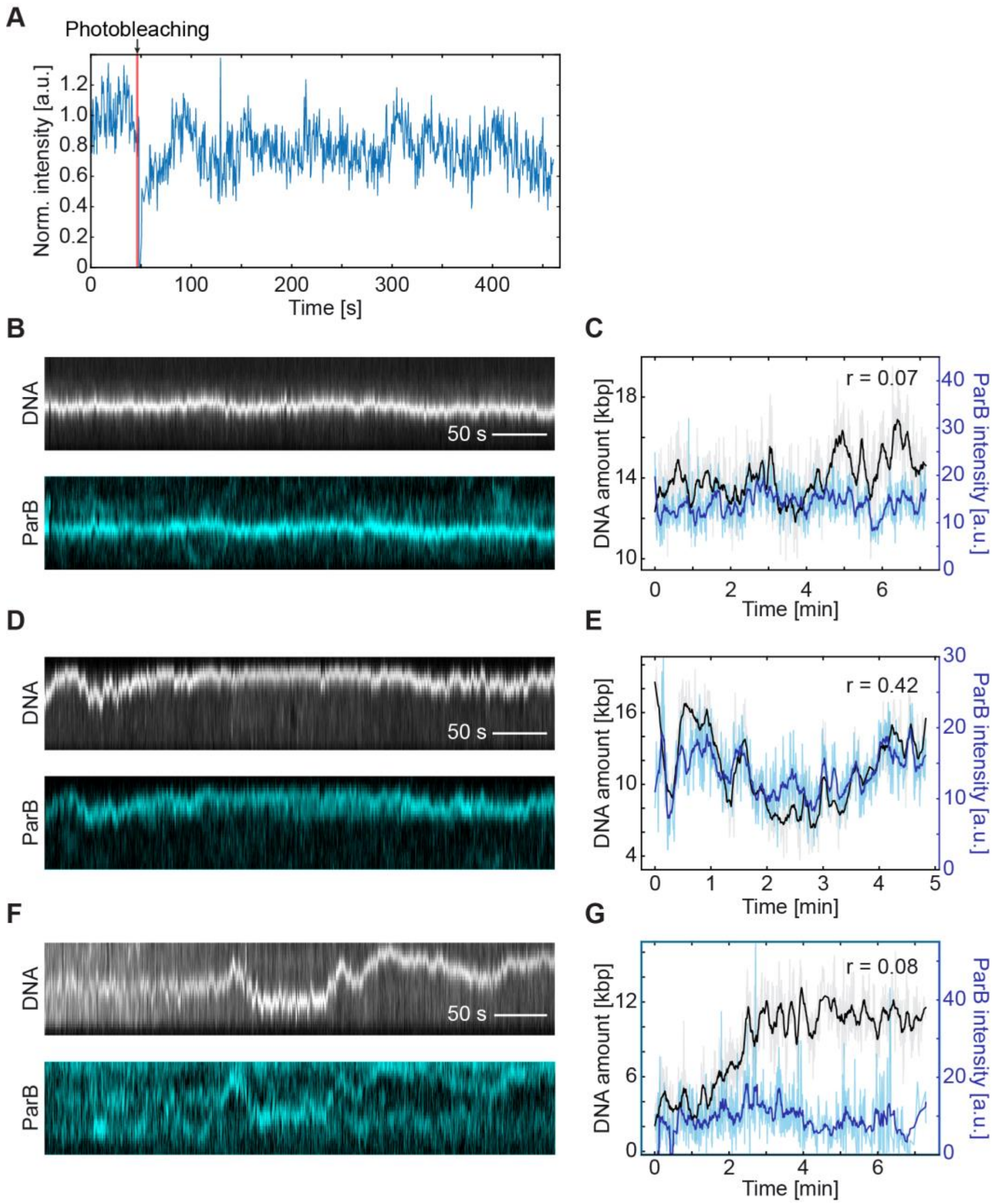

Figure S2. ParB:DNA clusters are dynamic and experience high ParB turnover. (A) Example FRAP (fluorescence recovery after photobleaching) fluorescence intensity plot of a single DNA$_{parS}$ molecule from Fig. 2A. The bleaching event is marked with red line. (**B)** Kymographs of DNA$_{parS}$ (top, grey) and ParB^{alexa647} (bottom, cyan) after the bleaching recovery events. (**C)** Quantification of the ParB signal intensity (light blue: raw data; purple: filtered data (Savitzky-Golay, time window =11 frames)) and the amount of DNA within the condensed region of the DNA$_{parS}$ (light gray: raw data; black: filtered data). Pearson correlation coefficient (r) shown in legend. (**D-E)** Same as (B) and (C) for an example molecule with a higher r value. (**F-G)** Same as (B) and (C) for an example molecule with a lower r value. Related to Figure 2.

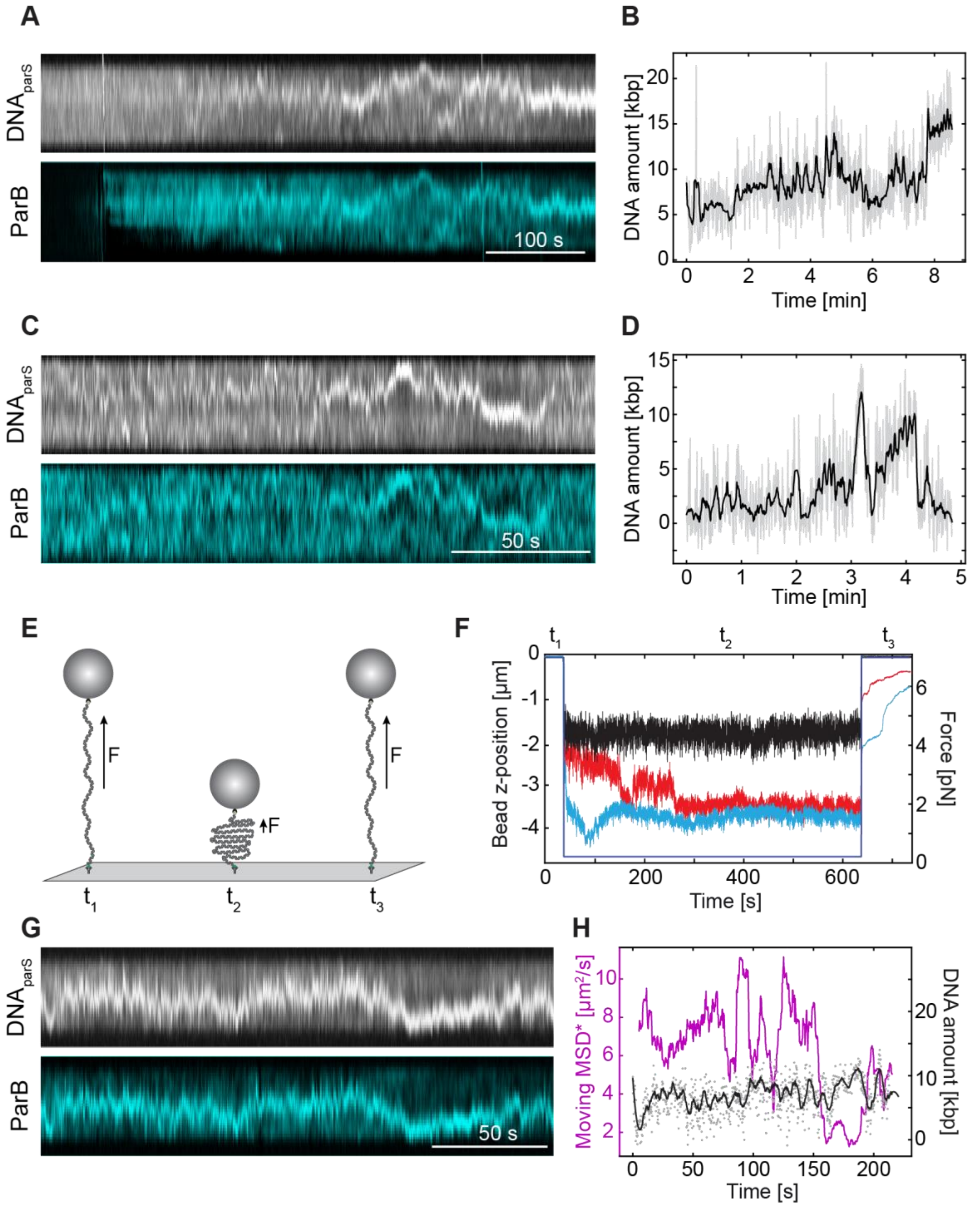

Figure S3. DNA$_{parS}$ molecules experience highly dynamic and fluctuating DNA condensation in the initial stages. (A) Time course experiment of ParB inflow and ParB-induced DNA condensation. Kymographs of DNA$_{parS}$ (top, grey) and ParB^{alexa647} (bottom, cyan) from the beginning of ParB loading up to dynamic DNA condensation. (**B)** Quantification of the amount of DNA within the condensed region of

the DNA$_{parS}$ (light grey: raw data; black: filtered data (Savitzky-Golay, time window =11 frames)). **C-D)** Same as in (A) and (B). (**E)** Time course experiment in MT assay. The DNA bead is lowered from 5 pN (t_1) to 0.3 pN for 5 min (t_2), and then back to 5 pN (t_3). **(F)** Magnetic bead z-position representing DNA$_{parS}$ extension in the presence of 25 nM ParB. Black trace without ParB does not show DNA condensation events, while in presence of ParB the DNA$_{parS}$ experiences diverse discrete condensation steps (blue, red). (**G)** Kymographs of DNA$_{parS}$ (top, grey) and ParB^{alexa647} (bottom, cyan) after ~20 min post ParB inflow, during the stable condensation stage. (**H)** Fluctuating DNA cluster position during a stable condensation. Magenta - apparent Mean Squared Displacement (MSD*, window size = 52 frames) of the ParB:DNA condensate tracked by the relative position along the 1D line of the tethered DNA. Gray dots – raw data of the DNA amount within the ParB:DNA condensate in each frame. Black – Savitzky-Golay filter (window size = 11 frames) of the DNA amount withing the condensate in shown in grey. Related to Figure 2.

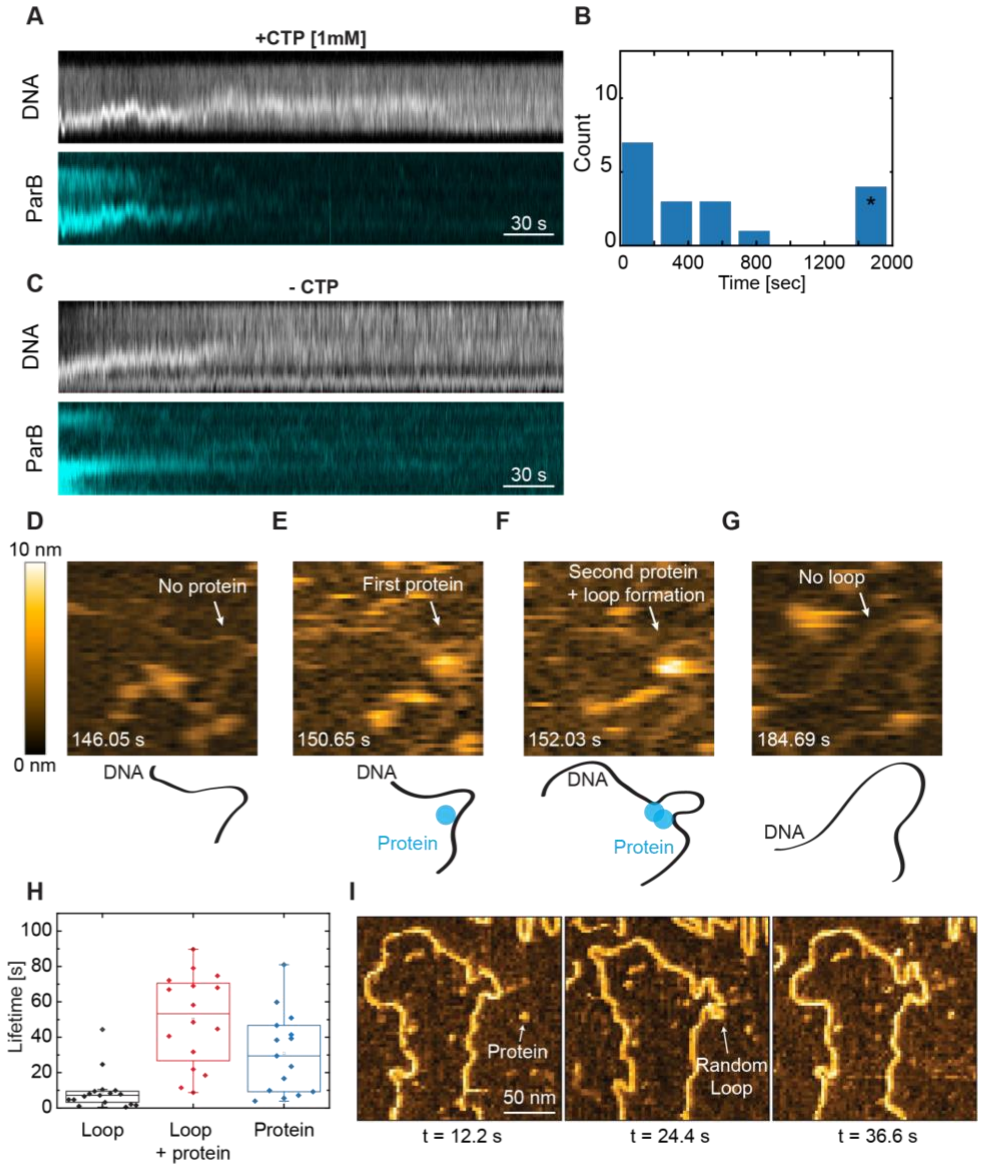

Figure S4. ParB-induced DNA condensation is reversible in the presence and absence of CTP.

(A) Kymographs of DNA$_{parS}$ (top, grey) and ParB^{TMR} (bottom, cyan) from the moment of buffer wash that does not contain ParB^{TMR} molecules. (**B)** Time from the beginning of the washing until the condensate entirely disassembles. Starred bin represents condensates that did not wash during the entirety of the video. (**C)** Same as (A) for the washing experiment using the buffer containing neither CTP nor ParB molecules. **(D-G)** High-speed AFM snapshots of transient DNA loop formation in the presence of ParB and CTP (7 nM; see Movie S1). Z-scale bar shown on the left. (**H)** Loop lifetimes on the DNA$_{parS}$ in the absence (black; mean±SD: 5.6±3.3 s; N = 15) and presence (red; mean±SD: 50±25 s; N = 16) of ParB molecules, and protein lifetime under the same conditions (blue; mean±SD: 30.8±22.9 s; N = 15). Related to Figure 3. **I)** High-speed AFM snapshots of transient DNA loop formation in the presence of ParB but in the absence of CTP.

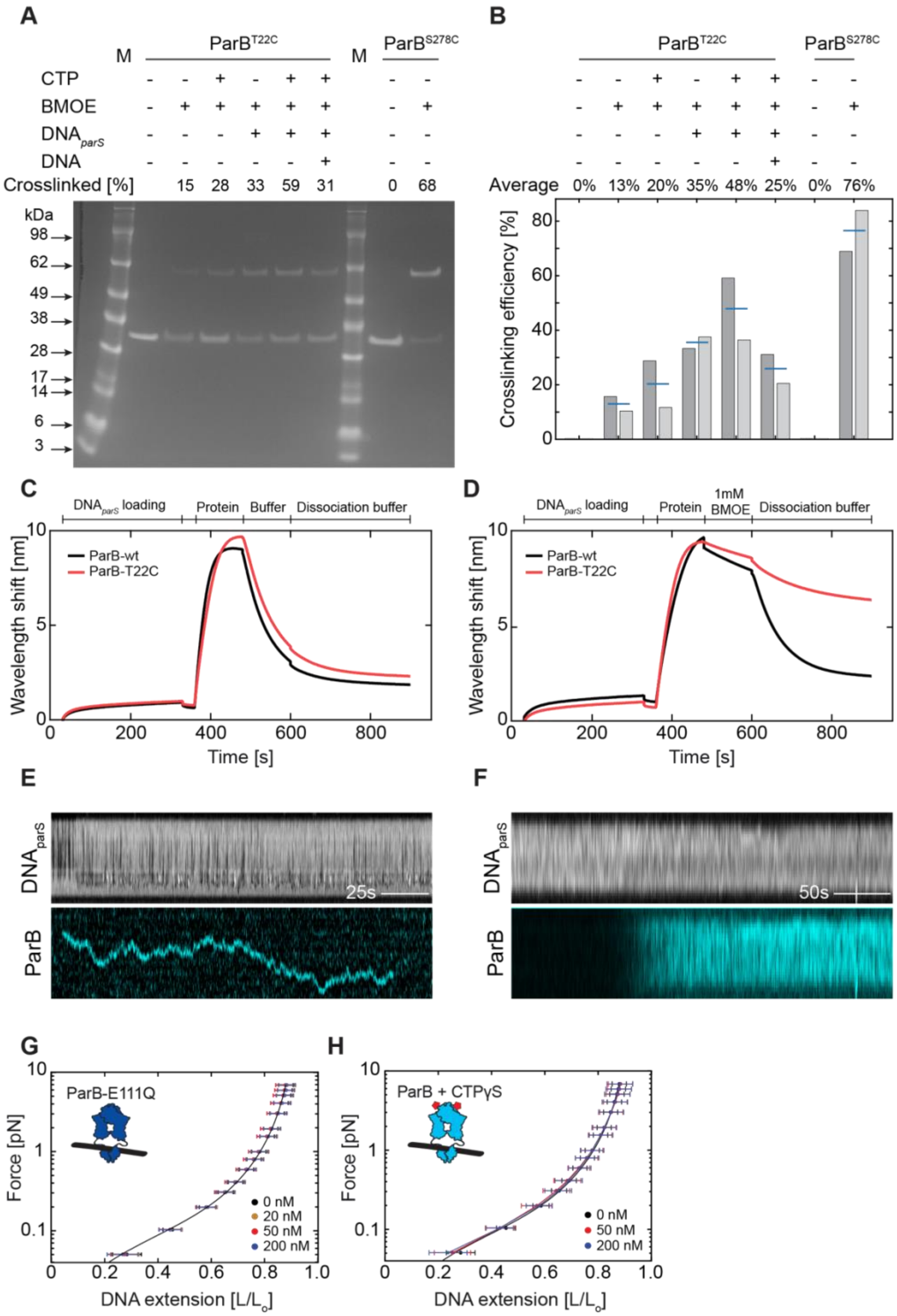

Figure S5. ParB mutants (T22C and S278C) can be crosslinked at their C-termini by BMOE.

(A) SDS-PAGE gel showing crosslinking efficiency of ParB$^{\mathbf{T22C}}$ (lanes 2-7) and ParB$^{\mathbf{S278C}}$ lanes (9-10) in the presence of 1 mM BMOE. Crosslinking efficiency is shown in the percentage of the total signal from both bands. Experimental conditions shown on top and more detailed in the Methods section. (**B)** Crosslinking efficiency from a duplicate experiment under the same conditions. Blue lines represent the

average crosslinked fraction from two experiments. (**C)** DNA loading and dissociation of ParB-wt and $\text{ParB}^{\mathbf{T22C}}$ (1 μM) on $\text{DNA}_{\textit{parS}}$ measured by BLI in the presence of 1 mM CTP (see Methods). During dissociation, an equivalent buffer lacking ParB protein and CTP was applied. **(D)** Same as in (C), with the additional step of introducing 1 mM BMOE for cysteine-crosslinking between the loading and dissociation step. **(E)** Kymographs showing $\text{DNA}_{\textit{parS}}$ (top) and single ParB dimer diffusing on the DNA (bottom). The final $\text{ParB}^{\text{Alexa647}}$ concentration was 0.2 nM. **(F)** Same as in E) but in the presence of 25 nM $\text{ParB}^{\text{Alexa647}}$. (**G)** Average DNA_{parS} extension (mean±SD) in MT in the presence of catalytically inactive $\text{ParB}^{\mathbf{E111Q}}$ mutant (N; 0 nM: 32, 20 nM: 36, 50 nM: 39, 200 nM: 36). **(H)** Same as in (G), but in the presence of the wild-type ParB protein with 1 mM CTPγS (N; 0 nM: 32, 50 nM: 33, 200 nM: 32).

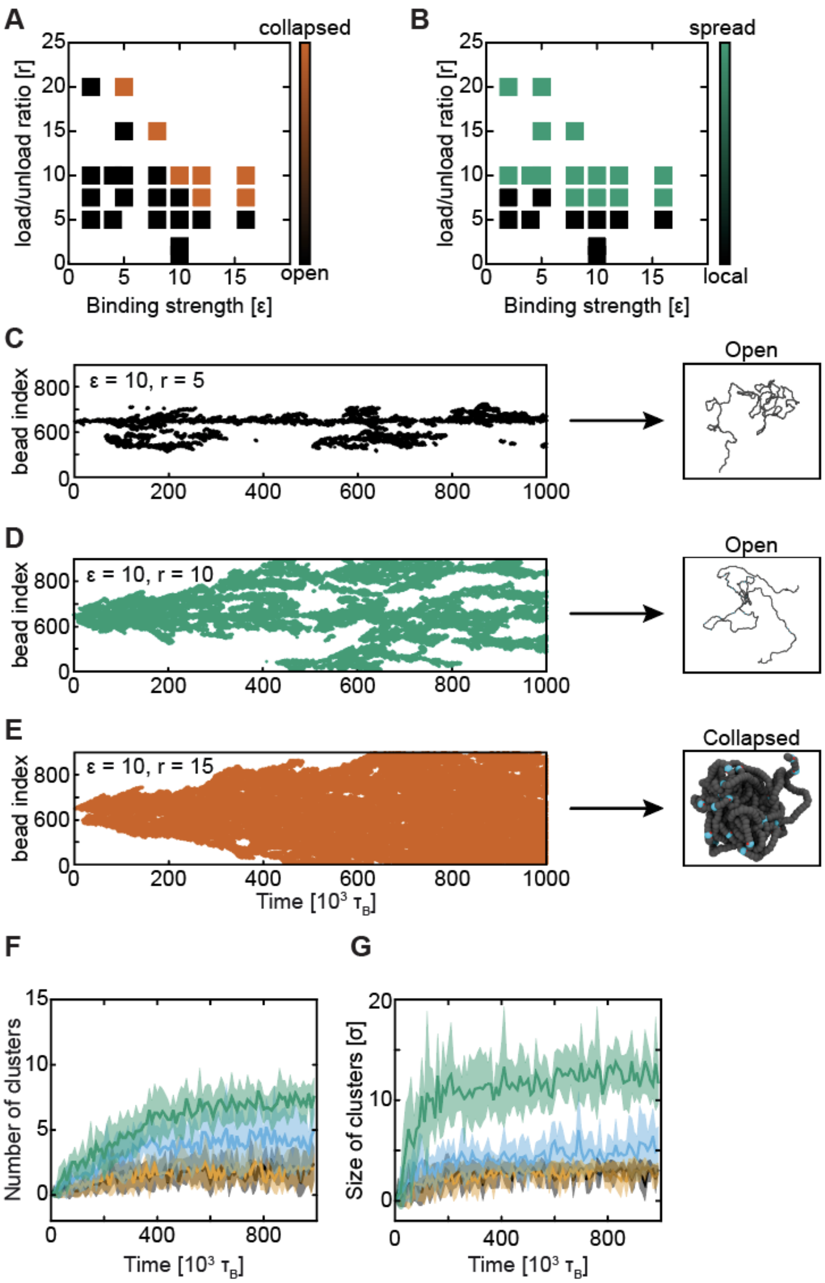

Figure S6. Efficient DNA condensation requires spreading and sufficient ParB interaction strength

(A) Final DNA polymer conformation (collapsed or open) in relation to load/unload rate (κ) and binding strength (epsilon). **(B)** ParB distribution over the DNA polymer (local or spread) in relation to load/unload rate (k) and binding strength (ε). **(C)** Left - Kymograph showing ParB distribution along the simulated DNA polymer at ε = 10 and κ = 5. Right – a snapshot of the final polymer conformation. **(D-E)** Same as (C) for k = 10 and κ = 15, respectively. **(F)** The number of DNA clusters over time per DNA. Legend shown on top. κ = 10. Shaded area is SD among 10 replicas of the same system with different initial configuration and seed. **(G)** The size of individual DNA clusters over time within a single DNA. Legend same as in F). κ = 10. Related to Figure 5.

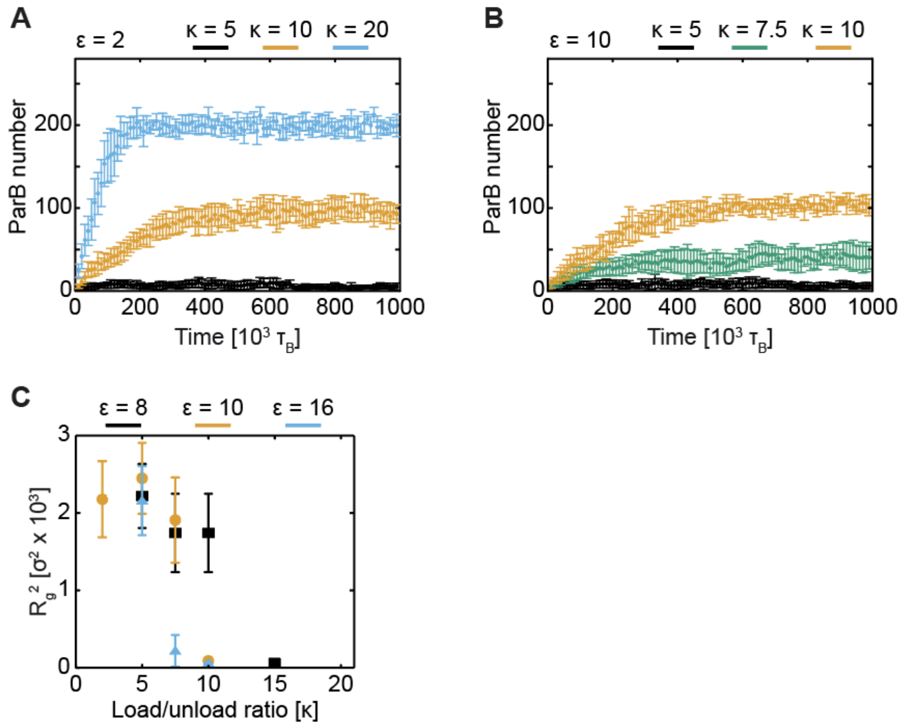

Figure S7. DNA-condensation by ParB experiences switch-like behavior due to a high ParB cooperativity.

(A) Total ParB numbers over time at increasing load/unload rates (κ) at a fixed interaction strength of ε = 2. (**B)** Same as (A) for ε = 10. **(C)** Radius of gyration of simulated DNA polymers as a function of κ. Related to Figure 5.

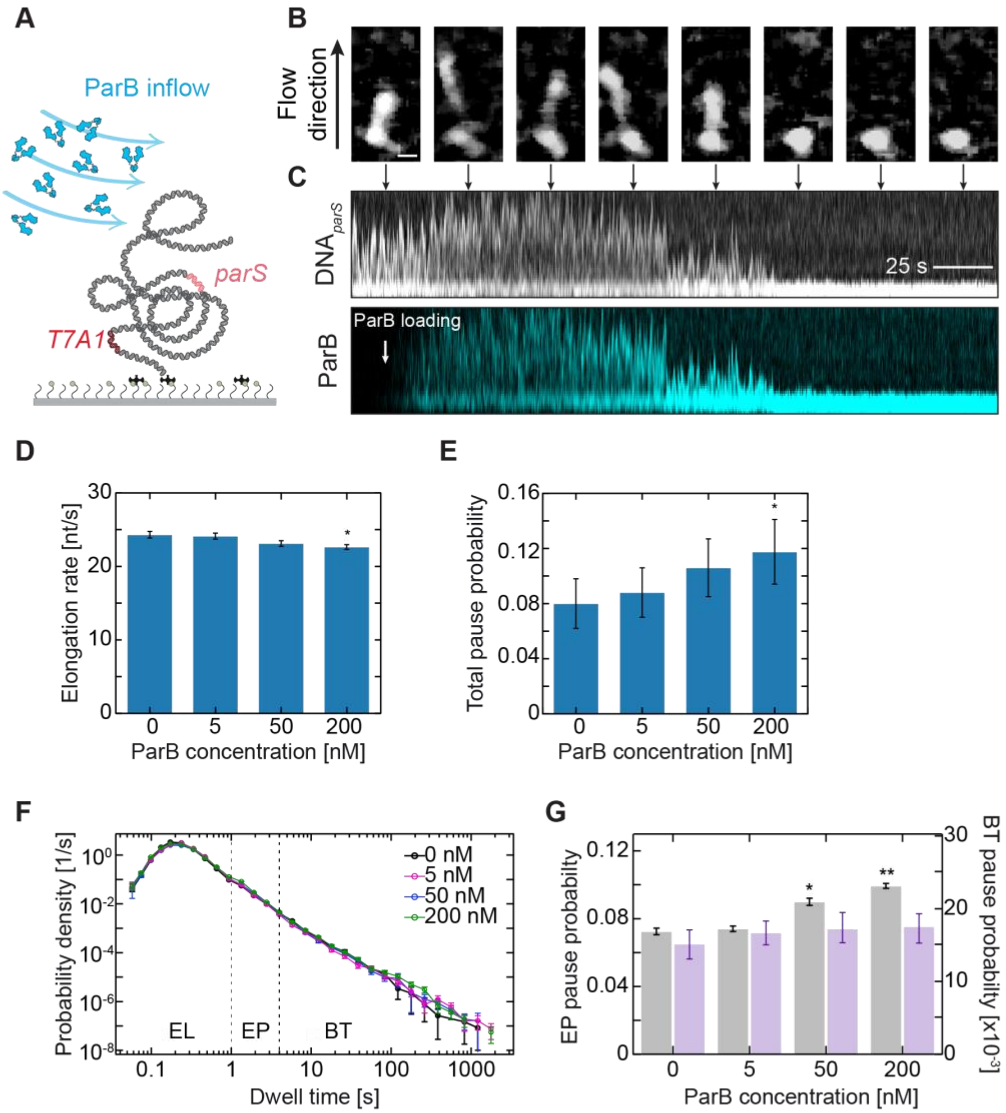

Figure S8. RNAP can efficiently transcribe DNA in the presence of ParB:DNA cluster

(A) Schematic representation of DNA$_{parS/T7A1}$ that is singly tethered to the glass surface via biotin-streptavidin interactions. **(B)** Snapshots of DNA $_{parS/T7A1}$ stained with SxO shown at different time points before and during the addition of 20 nM ParB. Scale bar = 1 µm. **(C)** Kymographs of DNA $_{parS/T7A1}$, stained with SxO (top) and ParB^{Alexa647} (bottom, 20 nM). White arrow indicates ParB loading onto DNA. **(D)** Pause-free elongation rate (mean±SD) and **(E)** total transcription pause probability (mean±SD) in the presence of increasing concentrations of ParB, extracted from Dwell time analysis in (C). (**F**) Superimposed dwell time distributions from RNAP trajectories in the presence of increasing concentrations of ParB (N; 0 nM: 27, 5 nM: 46, 50 nM: 29, 200 nM: 51). The distribution was constructed using the dwell times needed for RNAP to transcribe successive 5-bp segments of DNA. The dwell time distributions are separated with boundaries (see also Refs. 1,2) for the elongation region (0.1-1 s), short elemental pauses EP (1-5 s), and backtrack-induced pauses BT (>5 s). (**G**) The probability of elemental pause EP (grey) increases significantly with ParB concentration, while long backtrack pauses (purple) are not affected by ParB. Data was subject to statistical analysis using an unpaired, two-tailed t-test (p: ** <0.01; *< 0.05).

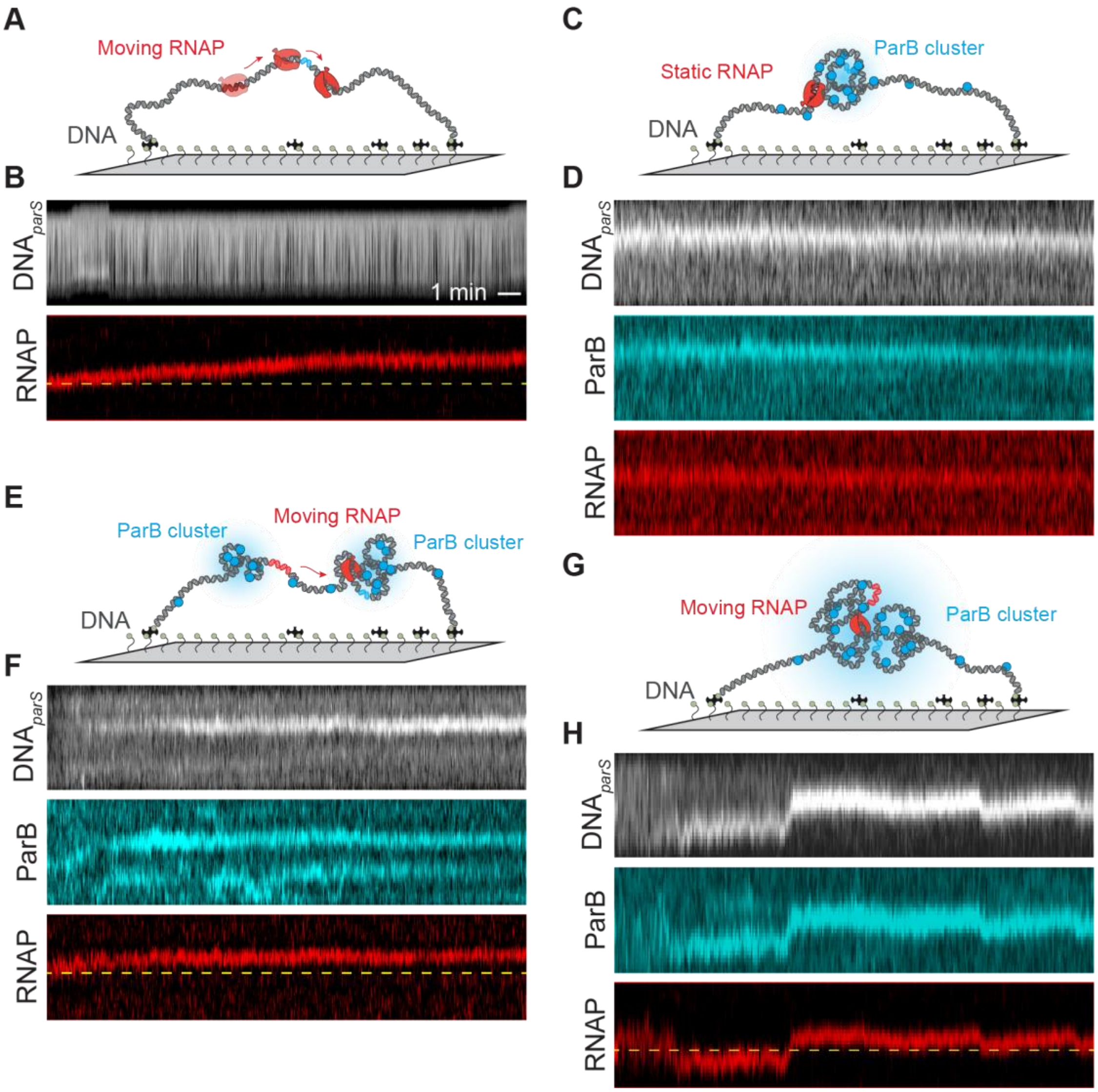

Figure S9. ParB proteins can efficiently condense the DNA in the presence of transcribing RNAP

(A) Schematic representation of RNAP transcription in our fluorescence assays **(B)** Kymographs of $DNA_{parS/T7A1}$ (top, grey) and transcribing $RNAP^{Alexa647}$ (bottom, red) from the moment of transcription initiation. Yellow dashed line represents initial location of the RNAP at the start of transcription **(C)** Schematic representation of ParB:$DNA_{parS/T7A1}$ cluster in presence of non-transcribing RNAP **(D)** Kymographs of $DNA_{parS/T7A1}$ (top, grey), $ParB^{TMR}$ (bottom, cyan) and non-transcribing/halted $RNAP^{Alexa647}$ (bottom, red). **(E)** Schematic representation of ParB:$DNA_{parS/T7A1}$ cluster in presence of transcribing RNAP at higher end-to-end length of $DNA_{parS/T7A1}$. **(F)** Same as (D). **(G)** Schematic representation of ParB:$DNA_{parS/T7A1}$ cluster in presence of transcribing RNAP at lower end-to-end length of $DNA_{parS/T7A1}$. **(H)** Same as (H). Related to Figure 6.

Table S1. Plasmids and cloning strategy used in this study for creation of the DNA constructs.

Plasmid Code	Plasmid Name		
#179	pBlueScript-GoldenGate-v2		
Cloning technique	Primers	Template	Template Origin
PCR (Q5 NEB E0555L)	JT492, JT 493	#64 pBlueScript-GoldenGate	Cees Dekker lab
KLD reaction	NEB M0554S		
Plasmid Code	Plasmid Name		
#64	pBlueScript-GoldenGate		
Cloning technique	Primers	Template	Template Origin
PCR (Q5 NEB E0555L)	JT290, JT291	#61 pBlueScript-noBsaI	Cees Dekker lab
KLD reaction	NEB M0554S		
Plasmid Code	Plasmid Name		
#61	pBlueScript-noBsaIsite		
Cloning technique	Primers	Template	Template Origin
PCR (Q5 NEB E0555L)	JT288, JT298	#18 pBlueScriptII SK+	Stratagene
KLD reaction	NEB M0554S		
Plasmid Code	Plasmid Name		
#66	pGGA-JT292JT293		
Cloning technique	Primers	Template	Template Origin
PCR (Q5 NEB E0555L)	JT492, JT493	Lambda	NEB N3011S
PCR (Q5 NEB E0555L)	JT403, JT401	pGGAselect	NEB N0309AAVIAL
Blunt ligation	Enzymes used: T4 PNK (NEB M0201), DPNI (NEB R0176), T4 DNA Ligase (NEB M0202)		
Plasmid Code	Plasmid Name		
#67	pGGA-JT294JT295		
Cloning technique	Primers	Template	Template Origin
PCR (Q5 NEB E0555L)	JT294, JT295	Template GeneBlock34	IDT Technologies
PCR (Q5 NEB E0555L)	JT403, JT401	pGGAselect	NEB N0309AAVIAL
Blunt ligation	Enzymes used: T4 PNK (NEB M0201), DPNI (NEB R0176), T4 DNA Ligase (NEB M0202)		
Plasmid Code	Plasmid Name		
#69	pGGA-JT310JT314JT311JT315		
Cloning technique	Primers	Template	Template Origin
PCR (Q5 NEB E0555L)	JT310, JT314	Lambda	NEB N3011S
PCR (Q5 NEB E0555L)	JT315, JT311	Lambda	NEB N3011S
PCR (Q5 NEB E0555L)	JT403, JT401	pGGAselect	NEB N0309AAVIAL

Gibson Assembly	NEB E5520 (NEB HiFi mix)		
Plasmid Code	Plasmid Name		
#163	pGGA-T7A1-ParS		
Cloning technique	Primers	Template	Template Origin
PCR (Q5 NEB E0555L)	JT472, JT473	#65 pGGA-JT298JT299	NEB N3011S
PCR (Q5 NEB E0555L)	JT466, JT467	#126 pSC-T7A1reverse+parS	NEB N3011S
Gibson Assembly	NEB E5529 (NEB HiFi mix)		
Plasmid Code	Plasmid Name		
#65	pGGA-JT298JT299		
Cloning technique	Primers	Template	Template Origin
PCR (Q5 NEB E0555L)	JT298, JT299	Template GeneBlock35	IDT Technologies
PCR (Q5 NEB E0555L)	JT403, JT401	pGGAselect	NEB N0309AAVIAL
Blunt ligation	Enzymes used: T4 PNK (NEB M0201), DPNI (NEB R0176), T4 DNA Ligase (NEB M0202)		
Plasmid Code	Plasmid Name		
#71	pGGA-JT312JT316JT313JT317		
Cloning technique	Primers	Template	Template Origin
PCR (Q5 NEB E0555L)	JT312, JT316	Lambda	NEB N3011S
PCR (Q5 NEB E0555L)	JT313, JT317	Lambda	NEB N3011S
PCR (Q5 NEB E0555L)	JT403, JT401	pGGAselect	NEB N0309AAVIAL
Gibson Assembly	NEB E5520 (NEB HiFi mix)		
Plasmid Code	Plasmid Name		
#126	pSuperCos-T7A1reverse+parS		
Cloning technique	Primers	Template	Template Origin
PCR (Q5 NEB E0555L)	MT236, MT239	#92 pSC-T7A1reverse	NEB N3011S
PCR (Q5 NEB E0555L)	MT235, MT238	#92 pSC-T7A1reverse	NEB N3011S
PCR (Q5 NEB E0555L)	MT234, MT237	#92 pSC-T7A1reverse	NEB N3011S
Gibson Assembly	NEB E5520 (NEB HiFi mix)		
Plasmid Code	Plasmid Name		
#92	pSC-T7A1reverse		
Cloning technique	Primers	Template	Template Origin
PCR (Q5 NEB E0555L)	JT405, JT406	#73 pIA146 T7A1	Irina Artsimovitch lab
Digest	#29 pSuperCos1-Lambda1,2	MscI	NEB R0534S
Blunt Ligation	Enzymes used: T4 PNK (NEB M0201), DPNI (NEB R0176), T4 DNA Ligase (NEB M0202)		
Plasmid Code	Plasmid Name		

#29 (from Ref. [3])	pSuperCos1-Lambda1,2		
Cloning technique	Primers	Template	Template Origin
Step 1			
PCR (Q5 NEB E0555L)	56-9,5kblambdafw, 57-9,5kblambdarev	Lambda	NEB N3011S
Digest	Digest PCR product with NotI + PspOMI, ligate in pSuperCos1 (Agilant) digested with NotI		
Step 2			
PCR (Q5 NEB E0555L)	58-5kblambdafw, 59-5kblambdarev	Lambda	NEB N3011S
Digest	Digest PCR product and plasmid made in step2 with BglII + BssHII		
Ligation	T4 DNA Ligase (NEB M0202)		

Plasmid Code	Plasmid Name		
#186			
Cloning technique	Primers	Template	Template Origin
PCR (Q5 NEB E0555L)	JT494, JT495	#18 pBlueScriptII SK+	Stratagene
KLD reaction	NEB M0554S		

Table S2. DNA primers used in this study for cloning and plasmid modification.

Primer name	Sequence
CD21	GACCGAGATAGGGTTGAGTG
CD22	CAGGGTCGGAACAGGAGAGC
JT138	CTGGCCTTTTGCTCACATGTTCTTTC
JT139	CAGATTTAGAAGGCAGATCACCAG
JT288	CGACAAGGTCTCGTCAGACGCGCCGGACGCTACCAGC
JT289	CGACAAGGTCTCGACTTCAAATCTTTCTGTATGAAGATTTGAGCACGT TGGCC
JT290	CGACAAGGTCTCGTCCACTAGTTCTAGAGCGG
JT291	CGACAAGGTCTCGTCGTATATCGATACCGTCGACCT
JT292	CGACAAGGTCTCGTCGTATATCGATACCGTCGACCTCGAGGGGCCCGG GCCCGGTACCCAATTC
JT293	CGACAAGGTCTCGTCCACTAGTTCTAGAGCGGCCGCGCCCGGGCACCG CGGTGGAGCTCCAGC
JT294	CGACAAGGTCTCCAGCCGCGACTTACCATGTAT
JT295	CGACAAGGTCTCCATACCGCGATGGTTGGAGTTCCA
JT298	CGACAAGGTCTCGTCAGACGCGCCGGACGCTACCAGC
JT299	CGACAAGGTCTCGACTTCAAATCTTTCTGTATGAAGATTTGAGCACGT TGGCC
JT310	TCGAGGGATCCGAATTCGAAGACTTGGTACCGACAAGGTCTCCGTATA TGAAAGAGACGACCACTGCCAG
JT311	TTCTGACGTCTTAATTAAGAAGACTACAGGCGACAAGGTCTCCCTGAC GCTGGCATTCGCATC
JT312	TCGAGGGATCCGAATTCGAAGACTTGGTACCGACAAGGTCTCGAAGT AAGAGACCTGGGCAGACAGGACTG
JT313	TTCTGACGTCTTAATTAAGAAGACTACAGGCGACAAGGTCTCGTGGAT CCGGGAGTGATTTCCGTCT
JT314	GCTAACCAATTCCTAGGCAGGTCATTG
JT315	CACTGTTGCCAATGACCTGCCTAG
JT316	TTCACATAAAACATTTTGCATCAGCGAC
JT317	GTGGTCAGGTTGTGGTGATTGGTC
JT337	AGAATAGACCGAGATAGGGTTGAGTG
JT338	GGCAGGGTCGGAACAGGAGAG
JT401	GTACCAAGTCTTCGAATTCGGATC
JT403	CCTGTAGTCTTCTTAATTAAGACGTCAG
JT405	TTCTGACGTCTTAATTAAGAAGACTACAGGCGACAAGGTCTCGACTTT ACAACTCACTATAAGGAGAGACAACTTAAAGAGAC
JT406	TAAATCTAACAAAATTCTATCCTGGGACATGCACTCTAGTCAGGATGA TGGTGATG
JT466	CGACTTTACAACTCACTATAAGGAGAGAC
JT467	GACTTACCATGTATCTCGTGCGGAAC
JT472	TGTCTCTCCTTATAGTGAGTTGTAAAGTCGGAAGTCGAGACCTTGTCG CCTGTAGTCTTC
JT473	GAGCGTTCCGCACGAGATACATGGTAAGTCTCTGACGAGACCTTGTCG GTACCAAGTCTTC
JT494	CTCGAGCTTCCCTTCCTTTCTCGCCACG
JT495	GCGGCCGCAGGCGGTAATACGGTTATCCACAGAATC
MT30	CTGCAGGAAGGTTTAAACGCATTTAGG

MT31	TAATACGACTCACTATAGGGAGACGC
MT32	CCTGTAGTCTTCTTAATTAAGACGTCAG
MT33	GTACCAAGTCTTCGAATTCGGATC
MT39	GATCCGAATTCGAAGACTTGGTACGGTCTCATCGTAAAGCTTCTTGATAACGGGGAC
MT40	GACGTCTTAATTAAGAAGACTACAGGGGTCTCAATGGTCCAGTCCCATTTCCCCTATCGC
MT234	CGCGGATGGAGTGAAAGAGATGCGCTA
MT235	TAGCGCATCTCTTTCACTCCATCCGCG
MT236	GCCCTCTGGTAAGGTTGGGAAG
MT237	GGCTTCCCAACCTTACCAGAGGGCTTGTTACACGTGGAACATGCCCCAGCTGGCAATTCCG
MT238	GGTTCGAAATGACCGACCAAGCACTAGTGACGCCCAACCTGCCATCACGAG
MT239	GCTTGGTCGGTCATTTCGAACC
56 9,5kblambdafw	AAGGAAAAAAGCGGCCGCTACATCTCGAGATGGTGCATCCCTCAAAACGAG
57 9,5kblambdarev	GGAAAGGGCCCGTAAAGTGATAATGATTATCATC
58 5kblambdafw	TTGGCGCGCTTGATACATCAACTGCACCTGATATTG
59 5kblambdarev	CCAGATCTACGACCTGCATAACCAGTAAG

Table S3. Synthetic DNA gene constructs used in this study.

Geneblock name	Sequence
Template GeneBlock34 (IDT)	
CCGCGACTTACCATGTATCTCGTGCGGAACGCTCACGTCTGCTCAGTGGGATGCCGGACAT<br>TACCGGACAACTGCTGCGGCACCTCAACTCCGATTTAATGAACGCAATATTCACAAGCAA<br>TGCGTGGTGTGCAACCAGCACAAAAGCGGAAATCTCGTTCCGTATCGCGTCGAACTGATT<br>AGCCGCATCGGGCAGGAAGCAGTAGACGAAATCGAATCAAACCATAACCGCCATCGCTG<br>GACTATCGAAGAGTGCAAGGCGATCAAGGCAGAGTACCAACAGAAACTCAAAGACCTGC<br>GAAATAGCAGAAGTGAGGCCGCATGACGTTCTCAGTAAAAACCATTCCAGACATGCTCGT<br>TGAAGCATACGGAAATCAGACAGAAGTAGCACGCAGACTGAAATGTAGTCGCGGTACGG<br>TCAGAAAATACGTTGATGATAAAGACGGGAAAATGCACGCCATCGTCAACGACGTTCTCA<br>TGGTTCATCGCGGATGGAGTGAAAGAGATGCGCTATTACGAAAAAATTGATGGCAGCAAA<br>TACCGAAATATTTGGGTAGTTGGCGATCTGCACGGATGCTACACGAACCTGATGAACAAA<br>CTGGATACGATTGGATTCGACAACAAAAAAGACCTGCTTATCTCGGTGGGCGATTTGGTT<br>GATCGTGGTGCAGAGAACGTTGAATGCCTGGAATTAATCACATTCCCCTGGTTCAGAGCT<br>GTACGTGGAAACCATGAGCAAATGATGATTGATGGCTTATCAGAGCGTGGAAACGTTAAT<br>CACTGGCTGCTTAATGGCGGTGGCTGGTTCTTTAATCTCGATTACGACAAAGAAATTCTGG<br>CTAAAGCTCTTGCCCATAAAGCAGATGAACTTCCGTTAATCATCGAACTGGTGAGCAAAG<br>ATAAAAAATATGTTATCTGCCACGCCGATTATCCCTTTGACGTATACGAGTTTGGAAAGCC<br>AGTTGATCATCAGCAGGTAATCTGGAACCGCGAACGAATCAGCAACTCACAAAACGGGAT<br>CGTGTGGCCACCAGGGGGCGCTAAAAGAAATCAAAGGCGCGGACACGTTCATCTTTGGTC<br>ATACGCCAGCAGTGAAACCACTCAAGTTTGCCAACCAAATGTATATCGATACCGGCGCAG<br>TGTTCTGCGGAAACCTAACATTGATTCAGGTACAGGGAGAAGGCGCATGAGACTCGAAAG<br>CGTAGCTAAATTTCATTCGCCAAAAAGCCCGATGATGAGCGACTCACCACGGGCCACGGC<br>TTCTGACTCTCTTTCCGGTACTGATGTGATGGCTGCTATGGGGATGGCGCAATCACAAGCC<br>GGATTCGGTATGGCTGCATTCTGCGGTAAGCACGAACTCAGCCAGAACGACAAACAAAAG<br>GCTATCAACTATCTGATGCAATTTGCACACAAGGTATCGGGGAAATACCGTGGTGTGGCA<br>AAGCTTGAAGGAAATACTAAGGCAAAGGTACTGCAAGTGCTCGCAACATTCGCTTATGCG<br>GATTATTGCCGTAGTGCCGCGACGCCGGGGGCAAGATGCAGAGATTGCCATGGTACAGGC<br>CGTGCGGTTGATATTGCCAAAACAGAGCTGTGGGGGAGAGTTGTCGAGAAAGAGTGCGG<br>AAGATGCAAAGGCGTCGGCTATTCAAGGATGCCAGCAAGCGCAGCATATCGCGCTGTGAC<br>GATGCTAATCCCAAACCTTACCCAACCCACCTGGTCACGCACTGTTAAGCCGCTGTATGAC<br>GCTCTGGTGGTGCAATGCCACAAAGAAGAGTCAATCGCAGACAACATTTTGAATGCGGTC<br>ACACGTTAGCAGCATGATTGCCACGGATGGCAACATATTAACGGCATGATATTGACTTATT<br>GAATAAAATTGGGTAAATTTGACTCAACGATGGGTTAATTCGCTCGTTGTGGTAGTGAGAT<br>GAAAAGAGGCGGCGCTTACTACCGATTCCGCCTAGTTGGTCACTTCGACGTATCGTCTGGA<br>ACTCCAACCATCGC	
Template GeneBlock35 (IDT)	
GCGCCGGACGCTACCAGCTTCTTTCCCGTTGGTGGGATGCCTACCGCAAGCAGCTTGGCCT<br>GAAAGACTTCTCTCCGAAAAGTCAGGACGCTGTGGCATTGCAGCAGATTAAGGAGCGTGG<br>CGCTTTACCTATGATTGATCGTGGTGATATCCGTCAGGCAATCGACCGTTGCAGCAATATC<br>TGGGCTTCACTGCCGGGCGCTGGTTATGGTCAGTTCGAGCATAAGGCTGACAGCCTGATTG<br>CAAAATTCAAAGAAGCGGGCGGAACGGTCAGAGAGATTGATGTATGAGCAGAGTCACCG<br>CGATTATCTCCGCTCTGGTTATCTGCATCATCGTCTGCCTGTCATGGGCTGTTAATCATTAC<br>CGTGATAACGCCATTACCTACAAAGCCCAGCGCGACAAAAATGCCAGAGAACTGAAGCTG<br>GCGAACGCGGCAATTACTGACATGCAGATGCGTCAGCGTGATGTTGCTGCGCTCGATGCA<br>AAATACACGAAGGAGTTAGCTGATGCTAAAGCTGAAAATGATGCTCTGCGTGATGATGTT<br>GCCGCTGGTCGTCGTCGGTTGCACATCAAAGCAGTCTGTCAGTCAGTGCGTGAAGCCACC<br>ACCGCCTCCGGCGTGGATAATGCAGCCTCCCCCCGACTGGCAGACACCGCTGAACGGGAT<br>TATTTCACCCTCAGAGAGAGGCTGATCACTATGCAAAAACAACTGGAAGGAACCCAGAAG	

TATATTAATGAGCAGTGCAGATAGAGTTGCCCATATCGATGGGCAACTCATGCAATTATTG
TGAGCAATACACACGCGCTTCCAGCGGAGTATAAATGCCTAAAGTAATAAAACCGAGCAA
TCCATTTACGAATGTTTGCTGGGTTTCTGTTTTAACAACATTTTCTGCGCCGCCACAAATTT
TGGCTGCATCGACAGTTTTCTTCTGCCCAATTCCAGAAACGAAGAAATGATGGGTGATGGT
TTCCTTTGGTGCTACTGCTGCCGGTTTGTTTTGAACAGTAAACGTCTGTTGAGCACATAGC
GCCCCCTGGTGGCCATCCTGTAATAAGCAGGGCCAGCGCAGTAGCGAGTAGCATTTTTTTC
ATGGTGTTATTCCCGATGCTTTTTGAAGTTCGCAGAATCGTATGTGTAGAAAATTAAACAA
ACCCTAAACAATGAGTTGAAATTTCATATTGTTAATATTTATTAATGTATGTCAGGTGCGA
TGAATCGTCATTGTATTCCCGGATTAACTATGTCCACAGCCCTGACGGGGAACTTCTCTGC
GGGAGTGTCCGGGAATAATTAAAACGATGCACACAGGGTTTAGCGCGTACACGTATTGCA
TTATGCCAACGCCCCGGTGCTGACACGGAAGAAACCGGACGTTATGATTTAGCGTGGAAA
GATTTGTGTAGTGTTCTGAATGCTCTCAGTAAATAGTAATGAATTATCAAAGGTATAGTAA
TATCTTTTATGTTCATGGATATTTGTAACCCATCGGAAAACTCCTGCTTTAGCAAGATTTTC
CCTGTATTGCTGAAATGTGATTTCTCTTGATTTCAACCTATCATAGGACGTTTCTATAAGAT
GCGTGTTTCTTGAGAATTTAACATTTACAACCTTTTTAAGTCCTTTTATTAACACGGTGTTA
TCGTTTTCTAACACGATGTGAATATTATCTGTGGCTAGATAGTAAATATAATGTGAGACGT
TGTGACGTTTTAGTTCAGAATAAAACAATTCACAGTCTAAATCTTTTCGCACTTGATCGAA
TATTTCTTTAAAAATGGCAACCTGAGCCATTGGTAAAACCTTCCATGTGATACGAGGGCGC
GTAGTTTGCATTATCGTTTTTATCGTTTCAATCTGGTCTGACCTCCTTGTGTTTTGTTGATGA
TTTATGTCAAATATTAGGAATGTTTTCACTTAATAGTATTGGTTGCGTAACAAAGTGCGGT
CCTGCTGGCATTCTGGAGGGAAATACAACCGACAGATGTATGTAAGGCCAACGTGCTCAA
ATCTTCATACAGAAAGATTTGAAGTAA

Supplementary movies

Movie S1. High-speed AFM movie of ParB loading and transiently looping the DNA molecule.

Movie S2. Molecular dynamics simulations of DNA polymer conformation in presence of ParB (ϵ = 5 and κ = 20) with dimer-of-dimers interaction (DoD).

Movie S3. Molecular dynamics simulations of DNA polymer conformation in presence of ParB (ϵ = 16 and κ = 20) with dimer-of-dimers interaction (DoD).

Movie S4. Molecular dynamics simulations of DNA polymer conformation in presence of ParB (ϵ = 2 and κ = 20) with multimer-of-dimers interaction (MoD). The polymer remains in the extended state even when ParB proteins are spread over the entire length of the polymer.

Movie S5. Molecular dynamics simulations of DNA polymer conformation in presence of ParB (ϵ = 10 and κ = 10) with multimer-of-dimers interaction (MoD). The polymer collapses in the condensed state maintained via transient ParB-ParB bridges.

SI references

1. Janissen, R., Arens, M.M.A., Vtyurina, N.N., Rivai, Z., Sunday, N.D., Eslami-Mossallam, B., Gritsenko, A.A., Laan, L., de Ridder, D., Artsimovitch, I., et al. (2018). Global DNA Compaction in Stationary-Phase Bacteria Does Not Affect Transcription. Cell *174*. 10.1016/j.cell.2018.06.049.
2. Janissen, R., Eslami-Mossallam, B., Artsimovitch, I., Depken, M., and Dekker, N.H. (2022). High-throughput single-molecule experiments reveal heterogeneity, state switching, and three interconnected pause states in transcription. Cell Rep *39*, 110749. 10.1016/j.celrep.2022.110749.
3. Burnham, D.R., Nijholt, B., De Vlaminck, I., Quan, J., Yusufzai, T., and Dekker, C. (2017). Annealing helicase HARP closes RPA-stabilized DNA bubbles non-processively. Nucleic Acids Res *45*, 4687-4695. 10.1093/nar/gkx147.
